# Altersepilepsie: Herausforderungen in Diagnostik und Therapie

**DOI:** 10.1007/s00391-021-01882-y

**Published:** 2021-04-23

**Authors:** Alexandra Rohracher, Eugen Trinka

**Affiliations:** Paracelsus Medizinische Privatuniversität Salzburg, Universitätsklinik für Neurologie, Ignaz-Harrer-Str. 79, 5020 Salzburg, Österreich

**Keywords:** Anfälle, Status epilepticus, Antiepileptika, Polypharmazie, Komorbiditäten, Seizures, Status epilepticus, Antiepileptic drugs, Polypharmacy, Comorbidities

## Abstract

Epilepsien stellen nach Demenzen und Schlaganfall die dritthäufigste neurologische Krankheitsgruppe bei älteren Menschen dar. Die Inzidenz der Epilepsien steigt im Alter, sodass aufgrund demografischer Entwicklungen in den kommenden Jahren mit einer weiteren Zunahme älterer Patienten mit Epilepsie zu rechnen ist. Die häufigsten Ursachen der Altersepilepsie stellen zerebrovaskuläre Erkrankungen und Demenzen dar. Anfälle bei älteren Menschen werden oft spät erkannt. Das Auftreten eines Status epilepticus ist bei älteren Patienten häufiger und mit erhöhter Morbidität und Letalität vergesellschaftet. Die medikamentöse Behandlung älterer Patienten wird durch Komorbiditäten und Polypharmazie erschwert, wobei Antiepileptika mit geringem Interaktionsprofil und guter Verträglichkeit zur Behandlung der Altersepilepsie gewählt werden sollten. Levetiracetam und Lamotrigin sind aufgrund geringer Interaktionen und guter Verträglichkeit Antiepileptika erster Wahl beim älteren Patienten.

## Abstract (en)

Epilepsy is the third most frequent neurological disorder in aged patients after stroke and dementia. The incidence of epilepsy increases with age with the highest rates in patients ≥ 65 years old. Due to demographic changes the number of aged patients with epilepsy is expected to increase further in the coming years. The leading cause of new onset epilepsy in aged patients is cerebrovascular disease followed by dementia. The recognition of seizures in aged patients is often delayed. Status epilepticus occurs more frequently in aged patients and is associated with a high mortality and morbidity. Antiepileptic drug (AED) treatment of aged patients is complicated by comorbidities and polypharmacy and AEDs with a low interaction profile and high tolerability should be selected. Levetiracetam and lamotrigine are the AEDs of choice due to low interactions and good tolerability.

## Lernziele

Nach der Lektüre dieses Beitrags

- können Sie die häufigsten Ursachen der Altersepilepsie nennen.
- erkennen Sie klinische Zeichen, die auf einen epileptischen Anfall bei älteren Patienten hindeuten können.
- kennen Sie wichtige Differenzialdiagnosen der Epilepsie und deren Diagnostik.
- können Sie grundlegende Überlegungen zur Wahl des geeigneten Antiepileptikums bei älteren Patienten anstellen.

## Einleitung

Epilepsien sind die dritthäufigste **neurologische Erkrankung**

neurologische Erkrankung

beim älteren Menschen [1] und mit einer Vielzahl sozioökonomischer Probleme vergesellschaftet. Epilepsie ist definiert als das Auftreten von mindestens 2 unprovozierten Anfällen im Abstand > 24 h oder Auftreten eines unprovozierten Anfalls und Vorliegen von Befunden, die die Wahrscheinlichkeit eines weiteren Anfalls auf > 60 % erhöhen [2]. Die Prävalenz der Epilepsie nimmt im Alter zu und steigt auf 1–2 % bei über 85-Jährigen [3, 4]. „Altersepilepsie“ wird meist als das Auftreten einer Epilepsie bei Menschen ≥ 60 Jahre definiert [5, 6]. Aufgrund der demografischen Entwicklungen ist mit einem weiteren Anstieg von Epilepsien zu rechnen. Neben der Betreuung von immer älter werdenden Patienten mit Epilepsie stellt somit insbesondere die Neudiagnose Epilepsie beim alten Menschen den behandelnden Arzt, den Patienten, Familie und Pflegepersonal vor eine Reihe von Herausforderungen in Diagnostik, Therapie und Management.

### Fallbeispiel

**Figure 1:**
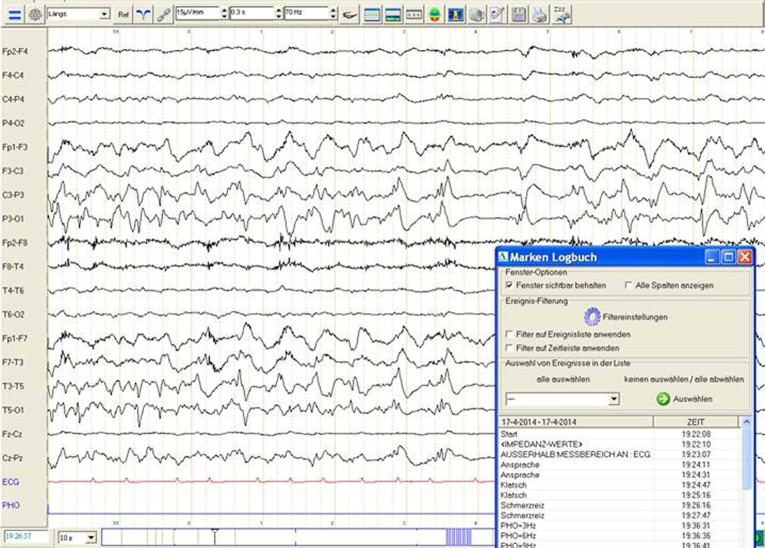

Eine 86-jährige Patientin wird aufgrund von Verwirrtheit mit der Rettung aus dem Seniorenheim in die Notfallambulanz transferiert. Hypertonie, Diabetes Typ 2 und Niereninsuffizienz sind vordiagnostiziert. Vor 2 Jahren hatte die Patientin einen linksseitigen ischämischen Mediainfarkt mit residualer Feinmotorikstörung und Wortfindungsstörungen. Am Aufnahmetag war die Patientin beim Frühstück unauffällig; eine halbe Stunde später fand sie die Pflegerin ohne Reaktion auf Ansprache, mit Blickwendung nach rechts und kauenden Mundbewegungen vor. Nach wenigen Minuten wurde die Patientin unruhig und befolgte keine Aufforderungen. Im Krankenhaus war sie wach, agitiert, ohne Paresen mit fehlender Sprachproduktion. In der Akut-CT des Schädels stellte sich ein linksseitiger temporaler ischämischer Defekt, ohne rezente Pathologie, dar. Das Akut-EEG (Elektroenzephalogramm) zeigte ein Statusmuster über links temporoparietal (Abb. 1). Durch die i.v.-Gabe von 2 mg Lorazepam und 1 g Levetiracetam konnte der Status durchbrochen werden. Die Patientin wurde zunehmend klarer, befolgte Aufforderungen, benannte Gegenstände korrekt und konnte nachsprechen. Die Diagnose eines Status aphasicus, ätiologisch residual („remote“) symptomatisch nach linksseitigem ischämischem Mediainfarkt, wurde gestellt und eine antiepileptische Therapie eingeleitet, worunter die Patientin anfallsfrei ist.

## Ursachen epileptischer Anfälle bei älteren Patienten

Die steigende Inzidenz und Prävalenz von Epilepsie im Alter lassen sich durch den demografischen Wandel und mit der Zunahme der zugrunde liegenden Ätiologien der Altersepilepsie erklären.

### Zerebrovaskuläre Erkrankungen und Altersepilepsie

Während bei jüngeren Patienten genetische Epilepsiesyndrome, Entwicklungsstörungen des Gehirns, Hippocampussklerose oder Hirntumoren die häufigsten identifizierbaren Ursachen sind, stehen bei alten Patienten Anfälle in Verbindung mit zerebrovaskulären Erkrankungen (ischämischer oder hämorrhagischer **Schlaganfall**

Schlaganfall)

an erster Stelle. Das Auftreten epileptischer Anfälle im Rahmen eines Schlaganfalls innerhalb von 7 Tagen (akut symptomatisch) oder mit zeitlicher Latenz nach stattgehabtem Schlaganfall (Remote oder residual symptomatisch) ist für beinahe die Hälfte aller Anfälle im Alter verantwortlich [8]. Bis zu 25 % aller Patienten erleiden nach einem Schlaganfall einen epileptischen Anfall [9]; das Risiko, eine Epilepsie zu entwickeln, beträgt 4 % nach einem Jahr und steigt nach 5 Jahren auf 8 % an [10]. Hämorrhagische Schlaganfälle und **intrazerebrale Blutungen**

intrazerebrale Blutungen

gehen mit einem noch höheren Risiko für epileptische Anfälle von 5–17 % einher [11]. Prädiktoren für das Auftreten epileptischer Anfälle nach ischämischem Schlaganfall sind ein kortikales Infarktareal, Infarkte im Versorgungsgebiet der A. cerebri media, eine arterioarterielle Ursache, schwere neurologische Ausfallsymptomatik (National Institutes of Health Stroke Scale [NIHSS] ≥ 11) und frühe Anfälle (< 24–48 h) [10]. Akut symptomatische Anfälle im Rahmen eines Schlaganfalls stellen häufig eine diagnostische Herausforderung in der Akutversorgung dar, da insbesondere eine **postiktale Lähmung**

postiktale Lähmung

(Todd-Parese) klinisch nicht von einer Parese im Rahmen einer frischen Ischämie unterschieden werden kann. Die Identifikation einer **akuten Durchblutungsstörung**

akuten Durchblutungsstörung

und deren umgehende Behandlung mithilfe der systemischen Lyse und/oder Thrombektomie im Falle des Verschlusses eines großen Gefäßes sind jedoch von größter Bedeutung und entscheidend für das Outcome.

Die Beziehung zwischen Epilepsie und zerebrovaskulären Erkrankungen ist nicht unidirektional. Das Risiko, einen Schlaganfall zu erleiden, ist bei Patienten mit spät beginnender Epilepsie erhöht (**„epilepsia praecursiva“**

„epilepsia praecursiva“,

[12]), wobei dies z. T. durch gemeinsame Risikofaktoren erklärt werden kann. Eine populationsbasierte Kohortenstudie in Finnland, die 245 Patienten mit Anfallsbeginn in der Kindheit und unkompliziertem Verlauf für 45 Jahre nachverfolgte, stellte fest, dass auch unabhängig von vaskulären Risikofaktoren MR-tomographische vaskuläre Läsionen in der Gruppe der Epilepsiepatienten signifikant häufiger waren [13]. Die engmaschige Kontrolle und Behandlung vaskulärer Risikofaktoren ist somit insbesondere bei älteren Epilepsiepatienten von großer Bedeutung.

#### Merke

- Zerebrovaskuläre Erkrankungen sind die häufigste zugrunde liegende Ursache der Altersepilepsie.
- Patienten mit Epilepsie im Alter haben auch im Fall keiner zerebrovaskulären Ursache der Epilepsie ein erhöhtes Risiko, einen Schlaganfall zu erleiden; Risikofaktoren sollten somit engmaschig kontrolliert werden.

#### Cave

Eine akute zerebrale Ischämie als Ursache eines akut symptomatischen Anfalls muss, insbesondere bei Fortbestehen einer Parese nach Sistieren des epileptischen Anfalls, mithilfe der zerebralen MRT ausgeschlossen werden, um keine wichtige Behandlungsoption zu verpassen.

### Demenz und Altersepilepsie

Eine zweite wichtige Ursache der Altersepilepsie stellen Demenzerkrankungen dar, die etwa 10–20 % der neu beginnenden Epilepsien zugrunde liegen [14]. Eine Fall-Kontroll-Studie in Großbritannien fand bei Patienten mit Demenz vom Alzheimer-Typ (**DAT**

DAT)

eine Inzidenzrate epileptischer Anfälle von 5,6/1000 Personenjahren und bei vaskulärer Demenz von 7,5/1000 Personenjahren. Im Vergleich hierzu lag die Inzidenzrate epileptischer Anfälle in der gleichaltrigen Kontrollgruppe ohne Demenz bei 0,8/1000 Personenjahren [15]. Die Prävalenz epileptischer Anfälle bei Patienten mit DAT beträgt etwa 5 % [16], und das Risiko, eine Epilepsie zu entwickeln, ist um das bis zu 10-Fache erhöht [17]. Die Beziehung zwischen Epilepsie und Demenz ist jedoch, noch mehr als beim Schlaganfall, bidirektional: Ältere Patienten mit neu diagnostizierter Epilepsie weisen bereits vor Therapiebeginn häufiger **kognitive Defizite**

kognitive Defizite

auf als gleichaltrige Kontrollpersonen [18] und haben ein 1,5-faches Risiko, innerhalb von 8 Jahren eine Demenz zu entwickeln [19]. Insbesondere bei Patienten mit **Temporallappenepilepsie**

Temporallappenepilepsie

(TLE) sind Gedächtnisprobleme ein häufig berichtetes Symptom, wobei bildgebende und neurophysiologische Studien gemeinsame Charakteristika von milder kognitiver Beeinträchtigung („mild cognitive impairment“, MCI) und Temporallappenepilepsie aufzeigen konnten [20]. Funktionelle Studien mithilfe der simultanen Elektroenzephalographie und des „functional magnetic resonance imaging“ (EEG-fMRI) fanden eine Abnahme der Konnektivität im „default mode network“ bei Patienten mit TLE [21], die auch bei Patienten mit MCI und DAT in ähnlicher Form beschrieben wurde [22] und negativ mit Kognition und Gedächtnisleistung assoziiert ist. Ein wesentlicher Faktor, der Epilepsie und Demenz verbindet, sind auch hier gemeinsame vaskuläre Risikofaktoren. Zudem wurden bei Patienten mit langjähriger Epilepsie signifikant häufiger **Amyloidablagerungen**

Amyloidablagerungen

im präfrontalen Kortex mithilfe der Amyloid-Positronen-Emissions-Tomographie nachgewiesen als bei gleichaltrigen Kontrollpersonen, was eine gemeinsame zugrunde liegende Pathophysiologie von Epilepsie und Demenz vermuten lässt [23]. Des Weiteren fand sich bei Patienten mit therapieresistenter TLE, die einem epilepsiechirurgischen Eingriff unterzogen wurden, in 94 % der Fälle (31/33 Patienten) **hyperphosphoryliertes Tau-Protein**

hyperphosphoryliertes Tau-Protein

in den Gewebsproben, wobei ein höheres Ausmaß der damit verbundenen Pathologie mit schlechterer kognitiver Leistung assoziiert war [24]. Es ist jedoch weiterhin Gegenstand intensiver Forschung, inwieweit gemeinsame zugrunde liegende Pathomechanismen die Entwicklung beider Erkrankungen bedingen, und welchen Einfluss Faktoren wie Anfallsfrequenz, Frequenz interiktaler epileptiformer Potenziale und Antiepileptikatherapie auf die Entwicklung kognitiver Defizite bei Epilepsiepatienten haben.

#### Merke

- Patienten mit Demenz, insbesondere DAT und vaskulärer Demenz, weisen ein erhöhtes Risiko auf, eine Epilepsie zu entwickeln, jedoch ist auch das Risiko der Entwicklung kognitiver Defizite und Demenz bei Epilepsiepatienten erhöht.
- Kognitive Einschränkungen sind insbesondere bei TLE häufig und oft der am stärksten die Lebensqualität einschränkende Faktor.

## Herausforderungen in der Diagnostik

Anfälle bei alten Menschen werden häufig erst spät erkannt [25], und die Rate an initialen Fehldiagnosen ist hoch [14]. Ein Grund hierfür ist die Semiologie, die sich von jüngeren Erwachsenen unterscheidet. Im Alter treten Anfälle oft in Form fokal nicht bewusst erlebter Anfälle mit Arrest, ohne klassische Symptome wie Automatismen oder psychische Phänomene, auf [26, 27, 28], und bilateral tonisch klonische Anfälle sind im Vergleich zu jungen Erwachsenen (65 %) seltener (26 %) [27]. Auren werden seltener berichtet und manifestieren sich oft unspezifisch in Form von Schwindel [27]. Symptome wie Innehalten oder eine häufig prolongiert bestehende **postiktale Desorientiertheit**

postiktale Desorientiertheit

[28] können z. B. als Verwirrtheitszustand im Rahmen einer Demenz fehlinterpretiert werden. Patienten selbst können teils aufgrund kognitiver Einschränkungen im Rahmen der zugrunde liegenden Erkrankungen wie Schlaganfall oder Demenz keine detaillierte Aussage über Symptome im Rahmen des Anfalls treffen. Die Diagnosestellung kann in diesen Fällen insbesondere bei zudem fehlender Fremdanamnese herausfordernd sein. Da alte Menschen häufiger allein leben und z. B. aufgrund eingeschränkter Mobilität teils weniger an gesellschaftlichen Ereignissen teilnehmen können, stellt **soziale Isolation**

soziale Isolation

einen weiteren erschwerenden Faktor in der korrekten Diagnosestellung dar [14].

Die Diagnose Epilepsie stellt alte Menschen vor eine Vielzahl psychosozialer Herausforderungen, da diese zu weiteren Einschränkungen von Selbstständigkeit und Mobilität z. B. durch Fahruntauglichkeit oder Angst vor Stürzen im Rahmen von Anfällen beitragen kann. Das Risiko **anfallsassoziierter Verletzungen**

anfallsassoziierter Verletzungen

durch Stürze ist bei alten Menschen hoch [29, 30], wobei Frakturen zu Krankenhausaufenthalten, weiterer Immobilisierung, Isolation und Langzeitmorbidität führen können.

### Wichtigste Differenzialdiagnosen

Eine der wichtigsten Differenzialdiagnosen epileptischer Anfälle beim alten Menschen stellen, wie auch bei jüngeren Patienten, Synkopen dar. Diese können meist durch gezielte Eigen- und Fremdanamnese mit speziellem Augenmerk auf Trigger-Faktoren (z. B. langes Stehen, körperliche Anstrengung, Hitze, Miktion etc.), Prodromalsymptomatik (ungerichteter Schwindel, Herzstolpern, Übelkeit etc.) und Dauer von epileptischen Anfällen differenziert werden. Neben **Reflexsynkopen**

Reflexsynkopen,

die die häufigste Ursache von Synkopen in jedem Lebensalter darstellen, muss bei alten Menschen aufgrund der häufig bestehenden kardialen Komorbiditäten eine kardiogene (arrhythmogene oder strukturelle) Genese ausgeschlossen werden. Insbesondere **arrhythmogene Synkopen**

arrhythmogene Synkopen

müssen aufgrund ihrer ungünstigen Prognose rasch erkannt und behandelt werden [31]. Die Durchführung eines Elektrokardiogramms (EKG) sowie, dem kardiovaskulären Risikoprofil entsprechend, die Anamnese (Palpitationen, Synkope bei körperlicher Anstrengung, Angina-pectoris-Symptomatik etc.) und Erhebung des klinischen Befunds, ergänzend eine Echokardiographie, sowie ggf. ein Langzeit-EKG sollten im Fall eines passageren Bewusstseinsverlustes erfolgen [31]. Synkopen, einhergehend mit motorischen Entäußerungen (**konvulsive Synkopen**

konvulsive Synkopen),

sind selten [32], können jedoch eine spezielle Herausforderung in der Differenzierung zu epileptischen Anfällen darstellen [33, 34]. Im Gegensatz zu bilateral tonisch klonischen Anfällen bestehen oft unspezifische Prodromi, gefolgt von einem Bewusstseinsverlust mit tonischer Extension der beidseitigen Arme und nachfolgend irregulären Zuckungen, die meist kürzer (20–30 s) andauern und mit rascher Reorientierung einhergehen. Die Fremdanamnese sollte insbesondere auf **lateralisierende Zeichen**

lateralisierende Zeichen

wie Herdblick, Kopfversion, einseitige motorische Entäußerungen und nachfolgende Verwirrtheit eingehen. Harnverlust kann auch bei konvulsiven Synkopen vorkommen, weist jedoch ebenso wie ein seitlicher Zungenbiss auf eine epileptische Genese hin. Laborchemisch zeigt sich nach epileptischen Anfällen meist ein Konzentrationsanstieg der **Kreatinkinase**

Kreatinkinase

(CK) als Folge der ausgeprägten motorischen Aktivität im Anfall.

Weitere wichtige Differenzialdiagosen epileptischer Anfälle stellen bei alten Menschen, aufgrund der klinischen Präsentationsform (Sprachstörung, Desorientiertheit, Areagibilität ohne motorische Phänomene) sowohl transitorisch ischämische Attacken, eine beginnende Demenz als auch ein Delir unterschiedlicher Ursache dar. **Basislaboruntersuchungen**

Basislaboruntersuchungen

mit Bestimmung von Blutzucker, Elektrolyten, Nieren- und Leberfunktionsparametern, einschließlich Ammoniak, bilden die Grundlage der Diagnostik. **Elektrolytstörungen**

Elektrolytstörungen

wie z. B. Hyponatriämien können sich insbesondere im Rahmen einer Diuretikatherapie oder antidepressiven Medikation mit selektiven Serotonin-Wiederaufnahmehemmern (SSRI) präsentieren. **Hypoglykämien**

Hypoglykämien

unter antidiabetischer Therapie sind bei älteren Patienten häufig und äußern sich in Form von fluktuierender Desorientiertheit oder Vigilanzschwankungen [35]. Auch **Intoxikationen**

Intoxikationen

sollten in der Akutdiagnostik ausgeschlossen werden. Eine akute Bildgebung ist in der Diagnostik unklarer Episoden mit Bewusstseinsbeeinträchtigung essenziell, um Pathologien mit fluktuierendem Verlauf wie z. B. Subduralhämatome unter oraler Antikoagulation etc. auszuschließen.

Die **Elektroenzephalographie**

Elektroenzephalographie

stellt in der Diagnostik epileptischer Anfälle, insbesondere innerhalb der ersten 48 h [36], zur Abgrenzung gegenüber Differenzialdiagnosen ein wichtiges diagnostisches Werkzeug dar. Allerdings gibt das erste EEG auch bei vorliegender epileptischer Genese nur in etwa einem Drittel der Fälle klare Hinweise in Form epileptiformer Potenziale. Unspezifische Veränderungen wie milde fokale Verlangsamungen oder diffuse Veränderungen sind bei älteren Patienten häufig und tragen nicht zur Klärung der Diagnose bei [14]. Bei ätiologisch unklarem **Delir**

Delir

ist die Durchführung eines EEG zum Ausschluss eines nichtkonvulsiven Status epilepticus (NCSE) indiziert.

Eine Bildgebung mithilfe der MRT nach Epilepsieprotokoll sollte auch bei älteren Patienten im Falle der Diagnose einer Epilepsie unklarer Ursache erfolgen. Der Ausschluss einer Autoimmunenzephalitis mithilfe der **Liquordiagnostik**

Liquordiagnostik

sollte entsprechend klinischer Präsentation und Bildgebungsbefund erwogen werden. Bei erstmaligem Auftreten von Anfällen im späten Erwachsenenalter, einhergehend mit kognitivem Abbau und Verhaltensauffälligkeiten, sowie Volumenvermehrung und Hyperintensität im Bereich der mesiotemporalen Strukturen (Amygdala, Hippocampus) in der zerebralen MRT sollte an dieses Krankheitsbild gedacht werden.

#### Merke

- Epileptische Anfälle beim älteren Patienten präsentieren sich häufig in Form fokal nicht bewusst erlebter Anfälle mit kognitiver Symptomatik (Aphasie, Verwirrtheit) und Arrest ohne prominente motorische Phänomene, sodass die Diagnosestellung oft verzögert erfolgt.
- Die Basisdiagnostik in der Abklärung unklarer passagerer Bewusstseinsstörungen umfasst neben der Labordiagnostik (einschl. Elektrolyten, Blutzucker, Nieren- und Leberfunktionsparametern) eine internistische Untersuchung (EKG, Blutdruck, ggf. Echokardiographie und Langzeit-EKG), eine Bildgebung des Schädels (akut mithilfe der CT, bei unklarer Befundlage und Verdacht auf epileptische Genese eine MRT nach Epilepsieprotokoll) und ein EEG.

### Status epilepticus

Bei älteren Menschen manifestieren sich Anfälle häufiger in Form eines SE, der als das Fortbestehen eines epileptischen Anfalls für die Dauer > 5 min beim bilateral tonisch klonischen Anfall (konvulsiver SE) und > 10 min beim fokalen SE mit oder ohne Bewusstseinsbeeinträchtigung (**NCSE**

NCSE)

definiert ist [37]. Ein Status epilepticus tritt bei älteren Patienten häufiger ohne vorbestehende Epilepsie de novo auf und präsentiert sich oft in Form eines NCSE mit oder ohne Koma [38]. Die Inzidenz des SE beträgt bei Menschen ≥ 60 Jahre 79,9/100.000 Erwachsene pro Jahr, verglichen mit 18,8/100.000 Erwachsene pro Jahr bei < 60-Jährigen [39]. Morbidität und Mortalität des SE sind hoch und steigen altersabhängig von 4,1 % bei < 60-Jährigen auf 22,5 % bei Patienten ≥ 60 Jahren an [38, 39].

#### Merke

- Der SE ist ein akuter neurologischer Notfall, der mit hoher Morbidität und Mortalität einhergeht und rasche medikamentöse Behandlung erfordert.

## Herausforderungen in der Therapie

Die medikamentöse Behandlung der Epilepsie bei alten Patienten stellt den behandelnden Arzt vor eine Reihe von Herausforderungen, wobei folgende Aspekte vor der Einleitung einer Antiepileptikatherapie beim alten Menschen bedacht werden müssen: (1) Alte Menschen reagieren meist sensibler auf Medikamente, insbesondere zentral wirksame Substanzen, wobei **unspezifische Nebenwirkungen**

unspezifische Nebenwirkungen

wie Schwindel, Müdigkeit, Gangunsicherheit und Konzentrationsstörungen häufig sind. Diese können im Weiteren zu Problemen wie sozialer Isolation, Sturzneigung mit Immobilisierung und Medikamenten-Non-Compliance führen, sodass Antiepileptika beim alten Menschen insbesondere hinsichtlich ihrer Verträglichkeit ausgewählt werden sollten. (2) Eingeschränkte Nierenfunktion, reduzierter hepataler Metabolismus und Mangelernährung mit verminderter Eiweißbindung führen zu Veränderungen von Pharmakokinetik und Pharmakodynamik beim alten Patienten. Die therapeutische Breite von Medikamenten ist bei alten Menschen häufig reduziert, sodass es oft zu **Überdosierung**

Überdosierung

kommt. (3) Aufgrund von Komorbiditäten nehmen alte Patienten häufig eine Reihe unterschiedlicher Medikamente ein, sodass das Risiko von **Medikamenteninteraktionen**

Medikamenteninteraktionen

hoch ist. Hiervon sind insbesondere Medikamente betroffen, welche durch das Zytochrom-P450-Enzym-System der Leber verstoffwechselt werden (z. B. orale Antikoagulanzien, Antiarrhythmika, Antidepressiva etc.), da eine Vielzahl von Antiepileptika zu einer Induktion dieser Enzyme führt (Carbamazepin [CBZ], Phenytoin [PHT], Phenobarbital [PB], Oxcarbazepin [OXC], Eslicarbazepinacetat [ESL], Topiramat [TPM]) oder Inhibition (Valproinsäure, VPA). (4) Die Wirkung von Medikamenten auf Lipidstoffwechsel, Reizleitungssystem des Herzens, Knochenstoffwechsel und Kognition etc. ist bei älteren, multimorbiden Patienten von entscheidender Bedeutung. Unerwünschte Effekte im Rahmen der Anwendung können schwerwiegende Folgen haben, z. B. Frakturen durch Reduktion der Knochendichte bei erhöhtem Sturzrisiko.

Vor Therapiebeginn sollte neben einer Basislabordiagnostik zur Beurteilung von Nierenfunktion und Lebersyntheseleistung auch eine **neuropsychologische Diagnostik**

neuropsychologische Diagnostik

erfolgen, um evtl. negative kognitive Effekte durch die medikamentöse Therapie beurteilen zu können. Im Allgemeinen sollte bei alten Patienten die antiepileptische Medikation in niedriger Dosierung begonnen und langsam gesteigert werden, wobei niedrigere Zieldosen als bei jüngeren Patienten bereits Wirksamkeit zeigen und angestrebt werden sollten [40, 41]. Die Bestimmung von Medikamentenspiegeln, die insbesondere bei älteren Antiepileptika vielerorts verfügbar sind, kann helfen, die minimal wirksame Zieldosis für jeden Patienten zu identifizieren und im Fall einer Abnahme der Nieren- oder Leberfunktion eine Dosisanpassung vorzunehmen.

### Merke

- Antiepileptika sollten bei alten Menschen vordergründig hinsichtlich der Verträglichkeit ausgewählt werden, wobei Medikamente mit Interaktionsrisiko sowie negativen Auswirkungen auf Stoffwechselleistung oder Kognition vermieden werden sollten.
- Das Credo ist auch in der Behandlung mit Antiepileptika beim alten Patienten „start low, go slow“, um die Verträglichkeit zu erhöhen, wobei auch geringere Zieldosen bereits eine ausreichende Wirksamkeit zeigen können.

### Häufig verwendete Antiepileptika: Carbamazepin, Lamotrigin, Levetiracetam

Einen Überblick über die pharmakokinetischen und pharmakodynamischen Eigenschaften der gebräuchlichsten Antiepileptika bietet Tab. 1.

**Table 1:** 

	Carbamazepin (CBZ)	Levetiracetam (LEV)	Lamotrigin (LTG)
Wirkmechanismus	Schnelle Inaktivierung spannungsabhängiger Natriumkanäle	Bindet an das synaptische Vesikelprotein SV2A	Inaktivierung spannungsabhängiger Natriumkanäle
Bioverfügbarkeit	Nahezu 100 %	100 %	100 %
Maximale Plasmakonzentration (C_max_)	Bei Retardpräparat nach 12 h (4–24 h)	Nach 1,3 h	–
Proteinbindung (%)	70–80	< 10	55
Verteilungsvolumen (l/kg)	0,8–1,9	0,5–0,7	0,92–1,22
Eliminations-HWZ	Nach Einzeldosis 36 h, durch Autoinduktion nach mehrfachen Gaben 16–24 h	7 ± 1 h Bei älteren Patienten um 40 % verlängert: 10–11 h	33 h (14–103 h)
Primäre Eliminationsroute	In der Leber durch Oxidation zum aktiven Metaboliten Carbamazepin-10,11-epoxid metabolisiert, Autoinduktion von CYP3A4 → raschere Elimination nach 3 bis 4 Wochen Behandlungsdauer 70 % renal, 30 % Fäzes	95 % unverändert renal	Glucuronidierung in der Leber zu inaktiven Metaboliten, Ausscheidung renal
Potenzielle Interaktionen	Starker Induktor von CYP3A4, zusätzlich Induktion von CYP1A2, CYP2C9, CYP2C19, UGT	LEV verringert Methotrexat-Clearance	VPA hemmt Glucuronidierung von LTG → erhöhte LTG-Wirksamkeit CBZ, PHT, orale Kontrazeptiva (Ethinylestradiol-Levonorgestrel-Kombinationspräparate) Rifampicin, Lopinavir/Ritonavir → reduzierte LTG-Wirksamkeit
Notwendigkeit der Dosisanpassung	Keine Dosisanpassung bei älteren Patienten	Bei älteren Patienten bis zu 40 % reduzierte Clearance Niereninsuffizienz: < 30 ml/min und 1,73 m^2^KOF 250–500 mg 2‑mal tgl. Bei Dialysepflichtigkeit 500–1000 mg einmal tgl. + 250–500 mg nach Dialyse	Keine Dosisanpassung bei älteren Patienten 50 %ige Reduktion bei Leberfunktionsstörung der Child-Pugh-Klasse B 75 % ige Reduktion bei Leberfunktionsstörung der Child-Pugh-Klasse C
Initialdosis	100–200 mg in 2 ED, Dosissteigerung um 100 mg/Woche	500 mg TD in 2 ED, nach 1 Woche 1000 mg TD	25 mg/Tag, Dosiserhöhung um 25 mg alle 2 Wochen **Cave**: bei Kombinationstherapie mit VPA Dosiserhöhung um 12,5 mg/Woche
Zieldosis	Max. 1200 mg	1000–(3000 mg)	100–200 mg
Besondere Nebenwirkungen	Hyponatriämie, Leukopenie > Thrombopenie, Schwindel, Diplopie, Kopfschmerzen, Ataxie, Tremor, Übelkeit *Allergische Reaktionen*: HLA-B*1502 (+HLA-A*3101) stellt einen Risikofaktor für das Auftreten allergischer Reaktionen dar (in der Han-chinesischen oder thailändischen Bevölkerung)	Reizbarkeit, Irritabilität, Konzentrationsstörungen	Allergische Reaktionen (Stevens-Johnson-Syndrom 1:1000) abhängig von der Geschwindigkeit der Aufdosierung

*CYP* Zytochrom-P450-System*, ED* Einzeldosis, *HLA* humanes Leukozytenantigen, *HWZ* Halbwertszeit, *PHT* Phenytoin, *TD* Tagesdosis, *UGT* Uridin-5´-diphospho-glucuronosyltransferase, *VPA* Valproat

Zwei multizentrische randomisierte kontrollierte Studien zur Beurteilung der Wirksamkeit und Tolerabilität von CBZ und **Lamotrigin**

Lamotrigin

(LTG) bei Patienten ≥ 65 Jahren [42, 43] zeigten, dass LTG bei vergleichbarer Wirksamkeit hinsichtlich Verträglichkeit dem CBZ in retardierter und unretardierter Form überlegen war. In einer rezenteren randomisierten doppelblinden Parallelgruppenstudie (STEP-ONE) waren CBZ in retardierter Form, LTG und **Levetiracetam**

Levetiracetam

(LEV) hinsichtlich ihrer Wirksamkeit vergleichbar, LEV und LTG schnitten hinsichtlich der Verträglichkeit gegenüber CBZ retard jedoch besser ab [44]. Eine weitere groß angelegte unverblindete, randomisierte Studie, die die „Standardantiepileptika“ CBZ retard bei fokalen Epilepsiesyndromen oder VPA bei generalisierten Epilepsiesyndromen mit LEV verglich (KOMET-Studie), fand in der Post-hoc-Analyse älterer Patienten ≥ 60 Jahre, dass bei vergleichbarer Wirksamkeit die Rate an Therapieabbrüchen unter LEV im Vergleich zu CBZ retard niedriger war [45].

Aufgrund der Notwendigkeit einer langsamen Eindosierung von LTG und lediglich oraler Darreichungsform ist insbesondere im Fall des Auftretens bilateral tonisch klonischer Anfälle oder eines SE auch bei älteren Patienten LEV als Medikament erster Wahl dem LTG überlegen. Zudem kann LEV im Gegensatz zu LTG und CBZ i.v. verabreicht werden und ist somit in der Akutbehandlung epileptischer Anfälle die Substanz erster Wahl. Levetiracetam wird zu 66 % unverändert renal ausgeschieden; bei älteren Patienten liegt selbst bei regelrechter Nierenfunktion eine durchschnittlich 40 % erniedrigte Clearance vor, sodass eine Dosisanpassung notwendig ist [46]. Unter antiepileptischer Therapie mit LEV wurden dosisabhängige **psychiatrische Nebenwirkungen**

psychiatrische Nebenwirkungen,

insbesondere Aggressivität und Stimmungsschwankungen bis hin zu psychotischen Störungen, beobachtet [47, 48, 49], die signifikant häufiger bei Patienten mit vorbestehender psychiatrischer Komorbidität auftraten [47]. Eine randomisierte placebokontrollierte doppelblinde Studie untersuchte den Einfluss von LEV, 1000 mg, vs. Placebo auf Kognition und Stimmung bei älteren Menschen > 65 Jahre, wobei sich eine geringe Zunahme von Irritabilität und Fatigue, jedoch kein negativer Effekt auf Kognition und Gedächtnis zeigte [50]. Im klinischen Alltag kann jedoch das Auftreten psychiatrischer Nebenwirkungen (Verhaltensauffälligkeiten, Reizbarkeit und depressive Stimmung) bei alten Patienten insbesondere bei höheren Dosen von LEV zu beobachten sein.

**Enzyminduzierende Antiepileptika**

Enzyminduzierende Antiepileptika

wie CBZ, zu einem geringeren Teil auch OXC und PHT, weisen das Risiko von Interaktion mit z. B. oralen Antikoagulanzien, Antiarrhythmika, Antidepressiva und Antihypertensiva bei Langzeitbehandlung auf. Daneben steigt das Risiko **metabolischer Nebenwirkungen**

metabolischer Nebenwirkungen.

Vaskuläre Risikofaktoren wie Lipidwerte und Homocystein steigen häufig unter der Therapie mit CBZ an, zudem fand sich unter der Langzeittherapie mit CBZ, PHT und VPA, nicht jedoch unter LTG, eine Zunahme der Intima-Media-Dicke. In einer Post-hoc-Analyse der STEP-ONE-Studie wurde eine reduzierte lipidsenkende Wirkung von Statinen unter Therapie mit CBZ nachgewiesen [51]. Somit sollten diese Antiepileptika bei Patienten mit bereits erhöhtem vaskulären Risikoprofil vermieden werden. Des Weiteren weisen **klassische Antiepileptika**

klassische Antiepileptika,

die über eine Blockade der spannungsabhängigen Natriumkanäle wirken (CBZ, OXC, PHT), das Risiko von Arrhythmien durch Interaktion mit dem kardialen Reizleitungssystem auf, sodass deren Einsatz bei Patienten mit kardialer Vorschädigung kritisch hinterfragt werden sollte. Elektrolytstörungen, insbesondere Hyponatriämien, treten sowohl unter Therapie mit CBZ als auch OXC auf und können insbesondere bei älteren Patienten in Kombination mit anderen Medikamenten wie Diuretika oder SSRI zu klinisch manifesten metabolischen Entgleisungen mit Verwirrtheit, Vigilanzminderung oder Anfällen führen [35].

Eine weitere klinisch relevante potenzielle Nebenwirkung enzyminduzierender Antiepileptika stellt die Störung des Knochenstoffwechsels mit Reduktion der **Knochendichte**

Knochendichte

dar. Insbesondere bei alten Patienten mit erhöhtem Frakturrisiko im Rahmen anfallsassoziierter Stürze oder multifaktorieller Gangstörung ist dies von großer Bedeutung.

#### Merke

- In der Behandlung älterer Patienten mit Epilepsie sind LEV und LTG dem CBZ retard bei vergleichbarer Wirksamkeit hinsichtlich der Verträglichkeit überlegen.
- Enzyminduzierende Antiepileptika (CBZ, OXC, PHT) sollten aufgrund ihres Interaktionsrisikos, negativer Auswirkungen auf vaskuläre Risikofaktoren sowie Knochenstoffwechsel bei älteren Patienten vermieden werden.

### „Neuere“ Antiepileptika: Lacosamid, Eslicarbazepinacetat, Perampanel und Brivaracetam

Die Entwicklung von Antiepileptika mit neuen Wirkmechanismen, geringerem Interaktionspotenzial und verbesserter Verträglichkeit hat in vergangenen Jahrzehnten zur Zulassung einer Reihe „neuer“ Antiepileptika geführt [52]. Die Datenlage zum Einsatz dieser Substanzen bei älteren Patienten ist i. Allg. gering, da Zulassungsstudien meist strenge Einschlusskriterien aufweisen und neben der Altersgrenze auch Komorbiditäten und Schwierigkeiten bei Rekrutierung und Einwilligungsfähigkeit den Einschluss dieser Patienten in randomisierte kontrollierte Studien limitieren.

**Lacosamid**

Lacosamid

(LCM) gehört zur Gruppe der Natriumkanalblocker; im Gegensatz zu CBZ, OXC und PHT verstärkt es die langsame Inaktivierung spannungsabhängiger Natriumkanäle. In einer Phase III-Non-Inferiority-Studie [53], die LCM mit CBZ retard bei Patienten ≥ 65 Jahren verglich, zeigten sich eine geringere Nebenwirkungsrate (LCM 35 % vs. CBZ retard 53 %), eine geringere Abbruchrate (LCM 21 % vs. CBZ retard 26 %) und bessere Wirksamkeit (Anfallsfreiheit LCM 73 % vs. CBZ-retard 60 %). Lacosamid wurde auch nach i.v.-Gabe aufgrund repetitiver Anfälle bei älteren Patienten ≥ 60 Jahren gut toleriert, wobei milde zentralnervöse Nebenwirkungen wie Müdigkeit, ungerichteter Schwindel und geringer Tremor berichtet wurden [54]. Aufgrund des seltenen Auftretens von atrioventrikulären (AV-)Überleitungsstörungen unter der Therapie mit LCM muss vor Therapiebeginn sowie nach Aufdosierung eine EKG-Untersuchung zum Ausschluss eines **AV-Blocks**

AV-Blocks

durchgeführt werden. Hinsichtlich Kognition und Einfluss auf die Knochendichte liegen keine ausreichenden Daten zur Therapie mit LCM bei älteren Patienten vor.

**Eslicarbazepinacetat**

Eslicarbazepinacetat

(ESL) ist eine „Tochtersubstanz“ von CBZ, die ihre Wirkung über die langsame Inaktivierung spannungsabhängiger Natriumkanäle entfaltet, jedoch im Gegensatz zu CBZ und OXC ein deutlich geringeres** Interaktionsrisiko**

Interaktionsrisiko

und nur eine geringe enzyminduzierende Wirkung aufweist. Eine multizentrische, nichtkontrollierte „Open-label“-Phase-III-Studie untersuchte Wirksamkeit und Verträglichkeit einer additiven Therapie mit ESL bei 72 Patienten ≥ 65 Jahren; in 65 % der Fälle traten Nebenwirkungen auf [55]. Ungerichteter Schwindel (12,5 %), Schläfrigkeit und Fatigue (8,3 %) sowie Hyponatriämien (8,3 %) wurden am häufigsten berichtet und führten bei 22 % der Patienten zum Therapieabbruch. Metabolische Störungen wie Dyslipidämien sind unter einer Therapie mit ESL geringer als unter CBZ-Anwendung, sodass ESL insbesondere aufgrund des niedrigeren Interaktionsrisikos Vorteile gegenüber der Muttersubstanz CBZ aufweist.

**Brivaracetam**

Brivaracetam

(BRV) ist eine dem LEV verwandte Substanz, die ebenso über das synaptische Vesikelprotein 2A wirkt, keine signifikante enzyminduzierende oder -inhibierende Wirkung aufweist und zum Großteil renal ausgeschieden wird. In einer Phase-IIb-Studie konnte eine Abnahme psychiatrischer Nebenwirkungen, die unter LEV bestanden, nach Umstellung auf BRV festgestellt werden [56], sodass diese Substanz evtl. bei psychiatrischen Nebenwirkungen unter LEV, jedoch guter Wirksamkeit als Alternative erwogen werden kann. Die Möglichkeit einer i.v.-Verabreichungsform ist insbesondere bei akuten Anfällen oder SE von großem Vorteil.

**Perampanel**

Perampanel

(PER) wirkt über eine nichtkompetitive Hemmung des glutamatergen α‑Amino-3-hydroxy-5-methyl-4-isooxazolpropionat(AMPA)-Rezeptors, die die Konzentration des primären exzitatorischen Neurotransmitters Glutamat im Zentralnervensystem reduziert. In eine multinationale, multizentrische Beobachtungsstudie zur Therapie mit PER wurden 135 Patienten ≥ 65 Jahren eingeschlossen [57], wobei sich eine Nebenwirkungsrate von 79 % zeigte. Ungerichteter Schwindel (24,7 %), Müdigkeit (16,5 %) und Verhaltensauffälligkeiten (16,5 %) waren die am häufigsten berichteten Nebenwirkungen, die in 6 % der Fälle zu einem Therapieabbruch führten. Die Einnahme der Medikation direkt vor dem Zubettgehen „an der Bettkante sitzend“, eine langsame Eindosierung mit Steigerung um 2 mg alle 4 Wochen sowie eine niedrige Zieldosis reduzieren Nebenwirkungen und steigern die Verträglichkeit. Ein Vorteil von PER ist die einmal tägliche Einnahme aufgrund einer langen **Halbwertzeit**

Halbwertzeit

von 105 h. Eine orale Lösung von PER ist seit Kurzem erhältlich, sodass die Verwendung auch bei Schluckstörung möglich ist.

Einen Überblick über die pharmakokinetischen und pharmakodynamischen Eigenschaften „neuer Antiepileptika“ bietet Tab. 2.

**Table 2:** 

	Eslicarbazepinacetat (ESL)	Brivaracetam (BRV)	Lacosamid (LCM)	Perampanel (PER)
Wirkmechanismus	Langsame Inaktivierung spannungsabhängiger Natriumkanäle Inhibition spannungsabhängiger Kalziumkanäle (Cav3.2)	Bindet an synaptisches Vesikelprotein (SV2A)	Langsame Inaktivierung spannungsabhängiger Natriumkanäle Bindet an CRMP‑2	Selektiver nonkompetitiver AMPA-Rezeptor Antagonist (antiglutamaterg)
Bioverfügbarkeit (%)	Oral > 90	Oral ≈ 100 I.v. 100	Oral ≈ 100 I.v 100	Oral ≈ 100
Maximale Plasmakonzentration (C_max_)	2–3 h	1 h (durch fettreiche Nahrung verzögert)	0,5–4 h, unabhängig von Nahrungsaufnahm	–
Proteinbindung (%)	30 46 % konzentrationsunabhängige Bindung an Blutzellen ↓ Bei älteren Patienten und Leberversagen, Niereninsuffizienz, Hypoalbuminämie	≤ 20 Reduziert bei älteren Patienten und Hypoalbuminämie	< 15 Reduziert bei älteren Patienten und Leberversagen, Niereninsuffizienz Hypoalbuminämie	95 Reduziert bei älteren Patienten und Leberversagen, Niereninsuffizienz, Hypoalbuminämie
Verteilungsvolumen (l/kg)	2,7 Erhöht bei älteren Patienten	0,5 Hohe Membranpermeabilität aufgrund der Lipophilie Erhöht bei älteren Patienten und Leberfunktionsstörung	0,5–0,8 Erhöht bei älteren Patienten, Leberfunktionsstörung und Hypoalbuminämie	1,1 Erhöht bei älteren Patienten, Leberfunktionsstörung und Hypoalbuminämie
Eliminations-HWZ	20–24 h *Keine* Autoinduktion	6–11 h Gering erhöht bei älteren Patienten	12–16 h Erhöht bei älteren Patienten mit 10- bis 35 %iger Zunahme der Plasmakonzentration > 65 Jahre	105 h Effektive HWZ 48 h Erhöht bei Leberfunktionsstörung (mild 306 h, mäßig 295 h)
Primäre Eliminationsroute	Hydrolytische Metabolisierung zum aktiven Metaboliten Eslicarbazepin; Metaboliten 90 % renal ausgeschieden	95 % renale Ausscheidung Metabolisierung durch Hydrolyse und Hydroxylierung, < 10 % unverändert renal	95 % renale Ausscheidung, teilweise unverändert und teilweise nach O‑Methylierung	30 % renal, 70 % hepataler Metabolismus durch CYP3A4 und CYP3A5
Potenzielle Interaktionen	Schwacher Induktor für CYP3A4 und UGT1A1 Inhibitor CYP2C19	Rifampicin senkt BRV-Serum-Spiegel um ca. 45 % BRV erhöht die Serumkonzentration von Carbamazepin-10, 11-epoxid	Enzyminduktoren senken Serumspiegel um 15–20 %	Enzyminduktoren reduzieren Serumspiegel bis zu 50 %: Clearance gesteigert durch PHT (1,7-fach), CBZ (2,7-fach), OXC (1,9-fach) PER erhöht OXC-Spiegel um 33 % Ketoconazol verlängert die HWZ um ≈ 15 % PER reduziert Levonorgestrel dosisabhängig (44 %)
Notwendigkeit der Dosisanpassung	Keine altersabhängige Dosisanpassung notwendig Bei Niereninsuffizienz: Kreatinin-Clearance 30–60 ml/min Initialdosis 200 mg Kreatinin-Clearance < 30 ml/min Anwendung nicht empfohlen	Keine altersabhängige Dosisreduktion notwendig Bei Leberfunktionsstörung Dosisreduktion, maximale TD 150 mg in 2 ED	Keine altersabhängige Dosisreduktion notwendig Bei schwerer Niereninsuffizienz mit Kreatinin-Clearance < 30 ml/min maximale TD 250 mg Bei mäßiger Leberfunktionsstörung maximale TD 300 mg Bei schwerer Leberfunktionsstörung nicht empfohlen	Keine Dosisreduktion bei älteren Patienten notwendig, jedoch bereits niedrigere Zieldosen wirksam Bei leichter bis mäßiger Leberfunktionsstörung maximale TD 8 mg Bei schwerer Leberfunktionsstörung nicht empfohlen
Initialdosis	400 mg einmal tgl. (abends)	50 mg/Tag in 2 ED	100 mg in 2 ED	2 mg einmal tgl., abends direkt vor dem Zubettgehen
Zieldosis	1200 mg einmal tgl.	200 mg/Tag in 2 ED	400–(600) mg	8–10 mg, bei älteren Patienten bereits 6 mg wirksam
Besondere Nebenwirkungen	Hyponatriämie, Verlängerung des PR-Intervalls, Gewichtszunahme, allergische Reaktionen: HLA-B*1502-Allel assoziiert mit erhöhtem Risiko eines Stevens-Johnson-Syndroms	Inappetenz, Reizbarkeit, Depression, Schwindel, Somnolenz	Schwindel, Kopfschmerzen, Diplopie, Übelkeit Verlängerung des PQ-Intervalls	Schwindel, Gangstörung, Müdigkeit, Reizbarkeit

*AMPA* α-Amino-3-hydroxy-5-methyl-4-isooxazolpropionat, *CBZ* Carbamazepin, *CRMP‑2* „collapsin response mediator protein 2“, *CYP* Zytochrom-P450-System*, ED* Einzeldosis, *HLA* humanes Leukozytenantigen, *HWZ* Halbwertszeit, *OXC* Oxcarbazepin, *PHT* Phenytoin, *TD* Tagesdosis, *UGT* Uridin-5´-diphospho-glucuronosyltransferase, *VPA* Valproat

#### Merke

- Neue Antiepileptika (LCM, ESL, BRV, PER) weisen teilweise andere Wirkmechanismen sowie ein geringeres Interaktionsrisiko auf, was zu besserer Verträglichkeit auch bei älteren Patienten führen kann.
- Die Datenlage zur Anwendung neuer Substanzen bei älteren Patienten, insbesondere im Hinblick auf Kognition, vaskuläre Risikofaktoren, Knochenstoffwechsel etc. ist noch begrenzt.
- Lacosamid ist ein bereits langjährig erprobtes, gut und rasch wirksames Antiepileptikum, das auch bei älteren Patienten gute Wirksamkeit und Verträglichkeit aufweist. Eine EKG-Untersuchung vor der Therapieeinleitung und im Verlauf sollte zum Ausschluss eines AV-Blocks erfolgen.

## Fazit für die Praxis

- Epileptische Anfälle sind beim älteren Menschen häufig und stellen oft das Symptom einer akuten zerebralen Schädigung z. B. im Rahmen eines Schlaganfalls dar. Das rechtzeitige Erkennen und die rasche Abklärung hinsichtlich zugrunde liegender Ätiologie sind für Behandlung und Prognose entscheidend.
- Zerebrovaskuläre Erkrankungen stellen die häufigste Ursache der Altersepilepsie dar, wobei neben der Anfallskontrolle eine gute Einstellung vaskulärer Risikofaktoren für das Therapieansprechen und Outcome von Bedeutung ist.
- Durch die klinisch oft nicht eindeutige Präsentation epileptischer Anfälle mit häufigem Fehlen motorischer Entäußerungen ist die korrekte Diagnosestellung erschwert. Bei Verdacht auf epileptische Anfälle sollten die rasche Zuweisung zu einem Neurologen und weitere Abklärung erfolgen, um die Einleitung einer adäquaten Therapie nicht zu verzögern.
- Die Diagnose der Epilepsie stellt für Patienten und Betreuungspersonen häufig eine große Herausforderung dar, und die adäquate Behandlung kann psychosoziale Probleme reduzieren.
- Die medikamentöse Therapie richtet sich bei älteren Patienten vordergründig nach Verträglichkeit und geringem Interaktionsrisiko. Eine niedrige Anfangsdosis, langsames Aufdosieren und das Anstreben der niedrigsten wirksamen Zieldosis steigern die Verträglichkeit und Lebensqualität.

## CME-Fragebogen

Welche der folgenden neurologischen Erkrankungen stellt die häufigste Ursache einer neu beginnenden Epilepsie im Alter dar?

Demenz vom Alzheimer-Typ

Idiopathisches Parkinson-Syndrom

Vaskuläre Demenz

Schädel-Hirn-Trauma

Schlaganfall

Ein 76-jähriger Patient wird aufgrund einer Bewusstseinsminderung, einhergehend mit Zuckungen des linken Arms und im Gesicht links, an die Notaufnahme transferiert. Bei dem Patienten ist eine Hypertonie vordiagnostiziert. Bei Eintreffen im Krankenhaus bestehen weiterhin Zuckungen des linken Arms und im Gesicht links, die nach Gabe von 2 mg Lorazepam sistieren. Nachfolgend ist der Patient somnolent, und es besteht eine Parese des linken Arms. Welche wichtige Ursache dieses erstmaligen fokalen Anfalls müssen Sie am dringlichsten ausschließen?

Hyponatriämie

Akuter rechtsseitiger Mediainfarkt

Intoxikation

Alkoholentzugsanfall

Demenz

Ein 81-jähriger Patient wird aufgrund einer plötzlich aufgetretenen Bewusstseinsminderung mit der Rettung in die Notaufnahme gebracht. In der Ambulanz kommt es zum Auftreten eines bilateral tonisch klonischen Anfalls mit Kopf- und Blickwendung nach rechts; nachfolgend ist er weiterhin bewusstlos, und es bestehen Zuckungen im Gesicht rechts fort. Welchen akut lebensbedrohlichen Notfall müssen Sie mithilfe des EEG ausschließen?

Katatoner Stupor

Nichtkonvulsiver Status epilepticus

Akut dekompensierte Demenz vom Alzheimer-Typ

Akinetische Krise bei M. Parkinson

Schlafapnoesyndrom

Eine 74-jährige Patientin stellt sich aufgrund 2‑maliger Episoden mit Bewusstseinsverlust in der Notaufnahme vor. Die Patientin schildert, Hitzegefühl und Herzrasen bemerkt und anschließend das Bewusstsein verloren zu haben. Sie sei, am Boden liegend, wieder zu sich gekommen, wobei sie sofort wusste, wo sie sich befand. Es bestand kein Zungenbiss oder Muskelkater. Eine Fremdanamnese liegt nicht vor. Klinisch präsentiert sich die Patientin ohne fokal neurologische Defizite. Blutdruck, Herzfrequenz und Temperatur sind unauffällig. Welche Ursache der 2‑maligen Bewusstseinsverluste müssen Sie in diesem Fall so schnell wie möglich ausschließen?

Psychogene nichtepileptische Anfälle

Kardiogene Synkopen

Elektrolytentgleisungen

Rezidivierende Transitorisch ischämische Attacken

Meningoenzephalitis

Welches der folgenden Symptome ist bei einem erstmals aufgetretenen epileptischen Anfall jenseits des 70. Lebensjahres am häufigsten?

Aura

Automatismen

Bilateral tonisch klonischer Anfall

Prolongierte Desorientiertheit

Motorische Phänomene

Ein 93-jähriger Patient wird aufgrund von Verwirrtheit in die Notaufnahme gebracht. Bei dem Patienten ist eine Niereninsuffizienz bekannt. Laut Tochter hätte der Patient aufgrund einer gastrointestinalen Infektion in den vergangenen Tagen kaum etwas getrunken. Klinisch besteht eine leichte Bewusstseinsbeeinträchtigung (Somnolenz), keine Paresen oder motorischen Entäußerungen; der Patient ist afebril. Welche der folgenden Untersuchungen zur Abklärung des Verwirrtheitszustandes leiten Sie als Erstes ein?

Magnetresonanztomographie des Schädels

Labordiagnostik, einschl. Nierenfunktionsparameter

Elektroenzephalographie

Neuropsychologische Testung

Liquoruntersuchung

Welches Antiepileptikum soll aufgrund der guten Wirksamkeit, Verträglichkeit, des geringen Nebenwirkungsprofils und der geringen Interaktionen als Medikament erster Wahl bei älteren Menschen zum Einsatz kommen (Evidenzklasse I)?

Carbamazepin

Perampanel

Levetiracetam

Lacosamid

Phenytoin

Welches der folgenden Antiepileptika hat die längste Eliminationshalbwertszeit?

Carbamazepin

Brivaracetam

Lacosamid

Levetiracetam

Perampanel

Warum sollte eine antiepileptische Therapie mit Carbamazepin bei älteren Menschen nur mit Vorsicht eingesetzt werden?

Als Enzyminduktor weist Carbamazepin eine Reihe von Wechselwirkungen mit anderen Medikamenten auf.

Carbamazepin ist ein nur schwach antiepileptisch wirksames Medikament.

Die Dosis von Carbamazepin muss bei älteren Menschen sehr hoch gewählt werden, um Wirksamkeit zu zeigen.

Carbamazepin ist kein geeignetes Medikament bei fokalen Epilepsiesyndromen.

Die Bestimmung des Medikamentenspiegels ist bei Carbamazepin nicht möglich, sodass eine Therapiekontrolle schwierig ist.

Welches ist das wichtigste Kriterium bei der Wahl eines geeigneten Antiepileptikums bei älteren Patienten?

Verfügbarkeit einer i.v.-Darreichungsform

Kurze Halbwertszeit

Möglichkeit der Bestimmung von Medikamentenspiegeln im Blut

Gute Verträglichkeit

Geringe therapeutische Breite
